# Fear, anxiety and depression in gastrointestinal stromal tumor (GIST) patients in the Netherlands: Data from a cross-sectional multicenter study

**DOI:** 10.1016/j.ijchp.2023.100434

**Published:** 2024-01-09

**Authors:** Deborah van de Wal, Dide den Hollander, Ingrid M.E. Desar, Hans Gelderblom, Astrid W. Oosten, Anna K.L. Reyners, Neeltje Steeghs, Olga Husson, Winette T.A. van der Graaf

**Affiliations:** a Department of Medical Oncology, The Netherlands Cancer Institute-Antoni van Leeuwenhoek, Amsterdam, the Netherlands; b Department of Medical Oncology, Radboud University Medical Center, Nijmegen, the Netherlands; c Department of Medical Oncology, Leiden University Medical Center, Leiden, the Netherlands; d Department of Medical Oncology, Erasmus MC Cancer Institute, Erasmus University Medical Center, Rotterdam, the Netherlands; e Department of Medical Oncology, University Medical Center Groningen, University of Groningen, Groningen, the Netherlands; f Department of Psychosocial Research and Epidemiology, The Netherlands Cancer Institute, Amsterdam, the Netherlands; g Department of Surgical Oncology, Erasmus MC Cancer Institute, Erasmus University Medical Center, the Netherlands

**Keywords:** Gastrointestinal stromal tumor, Psychological, Anxiety, Depression, Fear, Patient-reported outcome

## Abstract

**Background:**

This study aims to (1) investigate the prevalence of anxiety, depression and severe fear of cancer recurrence or progression in gastrointestinal stromal tumor (GIST) patients treated in a curative or palliative setting, (2) compare their prevalence with a norm population, (3) identify factors associated with anxiety, depression and severe fear, and (4) study the impact of these psychological symptoms on health-related quality of life (HRQoL).

**Methods:**

In a cross-sectional study, GIST patients completed the Hospital Anxiety and Depression Scale, Cancer Worry Scale, and EORTC QLQ-C30.

**Results:**

Of the 328 patients, 15% reported anxiety, 13% depression, and 43% had severe fear. Anxiety and depression levels were comparable between the norm population and patients in the curative setting, but significantly higher for patients in the palliative setting. Having other psychological symptoms was associated with anxiety, while current TKI treatment and anxiety were associated with depression. Severe fear was associated with age, female sex, palliative treatment setting, anxiety, and GIST-related concerns.

**Conclusion:**

GIST patients treated in a palliative setting are more prone to experience psychological symptoms, which can significantly impair their HRQoL. These symptoms deserve more attention in clinical practice, in which regular screening can be helpful, and appropriate interventions should be offered.

## Introduction

Psychological symptoms that cancer patients may experience vary from concerns, worries, sense of uncertainty, sadness and feelings of hopelessness, to specific psychiatric anxiety and depressive disorders (American Psychiatric Association & Association, 2013). Anxiety and depression are two of the most common psychological conditions, and fear of cancer progression (FCR) in cancer patients during active treatment and fear of cancer recurrence (FCR) in cancer survivors are common cancer-specific anxiety-related conditions (Grassi et al., 2023). In previous studies, experiencing anxiety, depression or FCR was associated with a diminished health-related quality of life (HRQoL), higher symptom burden (e.g., pain or nausea), poor treatment adherence, poorer prognosis and higher mortality (Arrieta et al., 2013; Carbajal-López et al., 2022; X. Wang et al., 2020, 2020b).

Gastrointestinal stromal tumor (GIST) patients also suffer from psychological symptoms, they emphasized experiencing fear of disease progression and resistance to treatment, fear of death, scan-related anxiety, and changes in mood and emotions including feeling down, depressed, and easily becoming emotional (de Wal et al., 2023; Fauske et al., 2020; Macdonald et al., 2012; van de Wal et al., 2023). GIST is a rare cancer that can arise anywhere along the gastrointestinal tract, affecting 8 per million persons per year (van der Graaf et al., 2018). Surgical resection is the cornerstone of treatment for localized GIST, combined with (neo)adjuvant imatinib in patients with locally advanced, sometimes large tumors at diagnosis, or at high risk of recurrence after their resection (Casali et al., 2022). In one in five patients the GIST has already metastasized to the peritoneum or liver at diagnosis (van der Graaf et al., 2018), these patients often depend on life-long treatment with tyrosine kinase inhibitors (TKIs), of which imatinib is the first line. Imatinib significantly improved the median survival of metastatic GIST patients from 12 to 68 months (Mohammadi et al., 2023). After failure of imatinib, sunitinib, regorafenib and ripretinib are currently registered (Casali et al., 2022). Despite these improvements in survival, most of the patients with metastatic GIST will eventually succumb to their disease (Blanke et al., 2008; Casali et al., 2017). For these patients the fear of disease progression, also described as the sword of Damocles, is undeniably a challenge (Custers et al., 2015). A Dutch study that assessed FCR in patients with localized or metastatic GIST, reported that half of the patients experienced severe fear resulting in more general and cancer-specific psychological distress compared to patients with low fear (Custers et al., 2015).

Up to now, most studies in GIST patients concerned patients in a metastatic setting, where it is more to be expected that patients experience fear and anxiety as they regularly undergo scans on which disease progression might be detected. However, the majority of GIST patients is treated in a curative setting, where surgery alone is curative in half of the patients, and 5-year relapse free survival rates are reaching 63–70% without or with adjuvant imatinib, respectively (Casali et al., 2015). In this group, it can be hypothesized that psychological symptoms (i.e., FCR, anxiety and depression) are less compared to patients treated in a palliative setting because they have a high chance of being cured and therefore the perspective of living a GIST-free life. Furthermore, imatinib itself might also result in psychological side effects, such as anxiety, being easily emotional or depression, since patients described this as being related to their imatinib treatment in qualitative studies (de Wal et al., 2023; Fauske et al., 2020; van de Wal et al., 2023), yet this has never been reported in larger quantitative studies. Therefore, the aims of this study were to (1) investigate the prevalence of anxiety, depression and severe FCR in GIST patients treated in a curative and palliative setting, (2) compare the prevalence of anxiety and depression with an age- and sex-matched norm population, (3) identify sociodemographic, clinical and psychological factors associated with anxiety, depression and severe FCR, and (4) study the impact of these psychological symptoms on health-related quality of life (HRQoL).

## Methods

### Study design & data collection

Data of the cross-sectional ‘Life with GIST’ study was used, which was approved by the medical ethical committee of the Radboud University Medical Center (2019-5888). The study design and data collection were described previously (van de Wal et al., 2023). In summary, this study was conducted among patients registered in the Netherlands Cancer Registry (NCR), diagnosed with GIST between 2008 and 2018, and treated within one of the five GIST reference centres. All patients provided informed consent, including permission to link their study data to data from the NCR. Data collection took place from September 2020 through June 2021 in the Patient-Reported Outcomes Following Initial treatment and Long-term Evaluation of Survivorship (PROFILES) registry (van de Poll-Franse et al., 2011).

### Sociodemographic and clinical characteristics

Patients self-reported sociodemographic (age, marital status, educational level) and clinical characteristics (co-morbidities via the Self-administered Co-morbidity Questionnaire (Sangha et al., 2003), tumor localization, treatment phase, and type of treatment). Additional (gender and socioeconomic status) and missing data were derived from the NCR database, if available.

### Psychological distress, anxiety and depression

The Hospital Anxiety and Depression Scale (HADS) (Olssøn et al., 2005) is a 14-item scale that was used to assess psychological distress, consisting of seven items on anxiety and seven items on depression. Each item was scored on a Likert scale ranging from 0 to 3. Patients’ symptoms were classified as ‘present’ (>11), ‘mild’ (8–10) or ‘no symptoms’ (0–7), for both subscales (Olssøn et al., 2005).

To compare the HADS data of our study sample to a norm population, HADS data of an age- and sex-matched normative sample without cancer was obtained from CentERdata, using a household panel representative of the population in the Netherlands. The panel members were randomly matched based on sex and age at the time of questionnaire completion. A total of 873 panel members were matched to 328 GIST patients (ratio 1:2.7).

### Cancer-related concerns

The Cancer Worry Scale (CWS) (Zebrack et al., 2006) is a 8-item scale to identify FCR, this scale was first validated for cancer survivors, but later also for GIST patients (Custers et al., 2015). Items were scored on a four-point Likert scale ranging from 1 to 4, scores were added up to calculate a total score, after which patients were classified as having ‘low fear’ (≤ 14) or ‘severe fear’ (≥ 14) (Custers et al., 2014). Because the CWS only addresses future recurrence and surgery, we added three GIST-specific items of own design to assess concerns of the need for TKIs in the future, dying from GIST in the near future and in the long term future. These items were rated on a four-point Likert scale as well, and patients were classified as either having concerns ‘yes’ (2–4) or ‘no’ (1).

### HRQoL

HRQoL was assessed by the European Organization for Research and Treatment for Cancer Quality of Life Questionnaire C30 version 3.0 (EORTC QLQ-C30) (Aaronson et al., 1993), which consists of 30 items assessing physical, role, cognitive, emotional, and social functioning, the financial impact, global quality of life, and specific symptoms (fatigue, nausea and vomiting, pain, dyspnea, insomnia, appetite loss, constipation, diarrhea). All items were scored on a 4-point Likert scale, except the items regarding global health and quality of life, which were scored from 1 (very poor) to 7 (excellent). Next, a linear transformation was conducted to standardize the raw scores of the scales, hence scores ranged from 0 to 100. Higher scores indicate a better global quality of life and functioning, whereas a higher symptom score indicates a higher symptom burden (Aaronson et al., 1993).

### Statistical analyses

All statistical analyses were performed using SPSS Statistics (IBM Corporation, version 29.0, Armonk, NY, USA). Two-sided *p*-values of <0.05 were considered statistically significant. Categorical data were described as frequencies and percentages, continuous data were described as mean and standard deviation (SD). Chi-square tests and independent *t*-test were conducted to compare sociodemographic and clinical characteristics, anxiety and depression scores, FCR and GIST-related concerns among GIST patients in a curative and palliative treatment setting. To compare anxiety and depression scores of GIST patients with the age- and sex-matched norm population, chi-square tests and ANOVA tests with post hoc Bonferroni were performed. To study the relationship between HRQoL and the outcomes FCR, anxiety and depression, we performed independent sample *t*-tests and ANOVA tests with a post-hoc Bonferroni correction. Three separate multivariable logistic regression analyses were conducted to examine the association between the outcomes severe FCR, symptoms of anxiety and symptoms of depression, and all variables (i.e., sociodemographic, clinical, psychological distress, cancer-related concern) with a *p*-value of <0.1 in the univariate logistic regression analysis. We then performed a backwards selection, removing the least significant variable from the model until all *p*-values were <0.1. Before each multivariable regression analysis, the included variables were checked for multi-collinearity using the variance inflation factors and variance proportions test.

## Results

In total, 521 GIST patients were invited to participate, of whom 328 (63%) responded. Our study population consisted of slightly more males (53%), had a mean age of 66.7 years at moment of completing the survey, and were on average 5.9 years after diagnosis (Table 1). Of the 328 patients, 260 (79.3%) patients were treated in a curative setting of whom 46 (17.7%) were currently on TKIs, and 68 (20.7%) patients were treated in a palliative setting of whom 67 were currently on TKIs. Groups did not differ significantly regarding sociodemographic characteristics. As expected, in the curative setting a significant higher percentage of patients received surgery (97.7% vs 64.7%, *p* = < 0.001), and in the palliative setting significantly more patients received TKI treatment (98.5% vs 55.8%, *p* = < 0.001).

**Table 1:** Sociodemographic and clinical characteristics of our study population.

	Total GIST sample (*n* = 328)	Curative setting (*n* = 260)	Palliative setting (*n* = 68)	*p*-value
Sex *n (%)*				
Male	174 (53.0)	139 (53.5)	35 (51.5)	.770
Female	154 (47.0)	121 (46.5)	33 (48.5)	
Age at survey completion *mean ± sd*	66.7 ± 10.4	66.6 ± 10.4	67.1 ± 10.2	.720
Socioeconomic status *n (%)*				
Low	150 (45.7)	113 (43.5)	37 (54.4)	.107
High	178 (54.3)	147 (56.5)	31 (45.6)	
Marital stage *n (%)*				
Married / Living with partner	246 (75.7)	193 (74.8)	53 (79.1)	.465
Not living with a partner	79 (24.3)	65 (25.2)	14 (20.9)	
Missing	3	2	1	
Educational level*n (%)				
Low/intermediate	206 (64.0)	165 (64.7)	41 (61.2)	.594
High	116 (36.0)	90 (35.3)	26 (38.8)	
Missing	6	5	1	
Comorbidity *n (%)*				
None	109 (33.4)	90 (34.7)	19 (28.4)	.384
1	71 (21.8)	58 (22.4)	13 (19.4)	
≥2	146 (44.8)	111 (42.9)	35 (52.2)	
Missing	2	1	1	
Time since diagnosis in years *Mean* *±* *SD*	5.9 ± 2.8	5.8 ± 2.8	6.0 ± 2.7	.676
Location primary GIST *n (%)*				
Stomach	207 (63.1)	173 (66.5)	34 (50.0)	.060⁎⁎
Small intestine	79 (24.1)	56 (21.5)	23 (33.8)	
Rectum	21 (6.4)	17 (6.5)	4 (5.9)	
Other	21 (6.4)	14 (5.4)	7 (10.3)	
Received TKI at some point	209 (64.3)	145 (55.8)	64 (98.5)	**<0.001**
Currently on TKI	110 (33.8)	46 (17.7)	64 (98.5)	**<0.001**
Had surgery for the GIST at some point	298 (90.9)	254 (97.7)	44 (64.7)	**<0.001**⁎⁎
Phase of treatment				
Declared cured, not in follow-up	61 (18.6)	61 (23.5)	–	
Not receiving active treatment, in follow up	153 (46.6)	153 (58.5)	–	
Receiving active treatment with curative intent	46 (14.0)	46 (17.7)	–	
Receiving active treatment with palliative intent	67 (20.4)	–	67 (98.5)	
Palliative setting without treatment	1 (0.3)	–	1 (1.5)	

Abbreviations: TKI = Tyrosine Kinase Inhibitor, SD = standard deviation.

⁎ Low (primary and secondary education), intermediate ((secondary) vocational education), and high (higher vocational education and academic education) educational level.

⁎⁎ Fisher's exact or likelihood ratio.

### Prevalence of anxiety, depression and severe fear

Of all GIST patients, 15% reported symptoms of anxiety, 13% symptoms of depression, and 43% had severe FCR. Severe FCR also occurred in patients that were not anxious or depressed, with 35.7% of these 253 patients reporting severe FCR. In comparison to patients in the curative setting, a significantly higher percentage of patients in the palliative setting reported symptoms of depression (26.5% vs 9.6%, *p* = .002), had severe FCR (73.4% vs 36.0%, *p* = < 0.001), and more often concerns about dying from GIST both in the near future (75.0% vs 28.9%, *p* = < 0.001) and in the long term future (84.4% vs 44.1%, *p* = < 0.001). In addition, patients in the palliative setting scored significantly higher on psychological distress (*M* = 10.1 vs *M* = 6.0, *p* = < 0.001), and had significant higher scores on the subscales anxiety and depression. An overview of the outcomes of the HADS, CWS and GIST-specific concerns is presented in Table 2.

**Table 2:** Outcomes of the HADS, CWS and GIST-specific concerns.

	Total GIST sample (*n* = 328)	Curative setting (*n* = 260)	Palliative setting (*n* = 68)	*p*-value
Total psychological distress score				
Mean ± SD	6.8 ± 6.6	6.0 ± 6.2	10.1 ± 7.1	**<0.001**
Total anxiety score				
Mean ± SD	3.6 ± 3.9	3.2 ± 3.6	5.2 ± 4.2	**<0.001**
Total depression score				
Mean ± SD	3.2 ± 3.2	2.8 ± 3.0	4.9 ± 3.4	**<0.001**
Symptoms of anxiety *n (%)*				
Present (>11)	20 (6.4)	14 (5.6)	6 (9.4)	.248*
Mild (8–10)	27 (8.6)	19 (7.6)	8 (12.5)	
No (0–7)	267 (85.0)	217 (86.8)	50 (78.1)	
Symptoms of depression *n (%)*				
Present (>11)	8 (2.5)	6 (2.4)	2 (3.1)	.002*
Mild (8–10)	33 (10.5)	18 (7.2)	15 (23.4)	
No (0–7)	273 (86.9)	226 (90.4)	47 (73.4)	
Fear of cancer recurrence or progression *n (%)*				
Severe fear	136 (42.9)	90 (35.6)	46 (71.9)	**<0.001**
Low fear	181 (57.1)	163 (64.4)	17 (28.1)	
Concerns about the need for TKI treatment in the future n (%)				
Yes	127 (40.4)	102 (40.6)	25 (39.7)	.890
No	187 (59.6)	149 (59.4)	38 (60.3)	
Concerns about dying from GIST in the near future *n (%)*				
Yes	121 (38.2)	73 (28.9)	48 (75.0)	**<0.001**
No	196 (61.8)	180 (71.1)	16 (25.0)	
Concerns about dying from GIST in the long term future *n (%)*				
Yes	166 (52.2)	112 (44.1)	54 (84.4)	**<0.001**
No	152 (47.8)	142 (55.9)	10 (15.6)	

⁎ Fisher's exact or likelihood ratio.

### Comparison with the norm population

Total psychological distress, anxiety, and depression scores of the GIST patients in the curative setting were comparable to the norm population, whereas GIST patients in the palliative setting scored significant higher on all three scales (Table 3). Furthermore, the percentage of GIST patients that experienced (mild) anxiety symptoms was higher, especially for those in the palliative setting, but not significantly higher. However, there was a statistically significant difference for depression symptoms, were a significant higher percentage of GIST patients in the palliative setting experienced mild symptoms.

**Table 3:** Comparison of HADS outcomes of GIST patients with an age- and sex-matched norm population.

	Norm population (*n* = 873)	Curative setting (*n* = 260)	Palliative setting (*n* = 68)	*p*-value*
Sex *n (%)*				
Male	463 (53.0)	139 (53.5)	35 (51.5)	.958
Female	410 (47.0)	121 (46.5)	33 (48.5)	
Age at survey completion *Mean ± SD*	64.9 ± 15.0	66.6 ± 10.4	67.1 ± 10.2	.121
Marital stage *n (%)*				
Married / Living with partner	549 (62.9)	193 (74.8)	53 (79.1)	**<0.001**
Not living with a partner	324 (37.1)	65 (25.2)	14 (20.9)	
Missing	–	2	1	
Educational level* *n (%)*				
Low/intermediate	558 (63.9)	165 (64.7)	41 (61.2)	.868
High	315 (36.1)	90 (35.3)	26 (38.8)	
Missing	–	5	1	
Comorbidity *n (%)*				
None	220 (25.2)	90 (34.7)	19 (28.4)	**.015**
1	252 (28.9)	58 (22.4)	13 (19.4)	
≥2	401 (45.9)	111 (42.9)	35 (52.2)	
Missing	–	1	1	
Total psychological distress score				
Mean ± SD	6.3 ± 5.9	6.0 ± 6.2	10.1 ± 7.1	**<0.001**⁎⁎
Post Hoc Bonferroni *p*-value	Ref	1.00	<0.001	
Total anxiety score				
Mean ± SD	3.2 ± 3.2	3.2 ± 3.6	5.2 ± 4.2	**<0.001**⁎⁎
Post Hoc Bonferroni *p*-value	Ref	1.00	<0.001	
Total depression score				
Mean ± SD	3.1 ± 3.3	2.8 ± 3.0	4.9 ± 3.4	**<0.001**⁎⁎
Post Hoc Bonferroni *p*-value	Ref	.578	<0.001	
Symptoms of anxiety *n (%)*				
Present (>11)	37 (4.2)	14 (5.6)	6 (9.4)	.062
Mild (8–10)	52 (6.0)	19 (7.6)	8 (12.5)	
No (0–7)	783 (89.8)	217 (86.8)	50 (78.1)	
Between group *p*-value	Ref	.406	.033	
Symptoms of depression *n (%)*				
Present (>11)	35 (4.0)	6 (2.4)	2 (3.1)	**<0.001**
Mild (8–10)	63 (7.2)	18 (7.2)	15 (23.4)	
No (0–7)	774 (88.8)	226 (90.4)	47 (73.4)	
Between group *p*-value	Ref	.453	<0.001	

⁎ *p*-value of the Chi-square test or ANOVA.

⁎⁎ Because the ANOVA was significant, a post hoc Bonferroni correction was performed.

### Factors associated with anxiety, depression and severe fear

For our uni- (Supplementary Table A1-A3) and multivariable (Table 4) logistic regression analysis the total GIST population was analyzed. Experiencing symptoms of depression (OR 19.7; 95% CI 8.2–47.2; *p* = <0.001), severe FCR (OR 2.9; 95% CI 1.2–6.9; *p* = .016) and having concerns about the need for TKI treatment in the future (OR 2.5; 95% CI 1.1–5.6; *p* = .031) were associated with higher odds of experiencing anxiety symptoms. While being currently on TKIs (OR 3.2; 95% CI 1.3–7.5; *p* = .009) and having symptoms of anxiety (OR 24.2; 95% CI 10.3–56.9, *p* = < 0.001) were associated with higher odds of experiencing symptoms of depression. Being female (OR 2.2; 95% CI 1.2–4.1; *p* = .012), receiving treatment in a palliative setting (OR 2.3; 95% CI 1.2–5.1; *p* = .032), experiencing symptoms of anxiety (OR 3.8; 95% CI 1.5–9.9; *p* = .006), having concerns about the need for TKI treatment in the future (OR 2.4; 95% CI 1.3–4.4; *p* = .008), dying from GIST in the near future (OR 4.4; 95% CI 2.1–9.3; *p* = <0.001) and in the long term future (OR 3.0; 95% CI 1.5–6.4; *p* = .003) were associated with higher odds of having severe FCR, whereas older age (OR 0.95; 95% CI 0.92–0.98; *p* = .001) was associated with lower odds of experiencing severe FCR.

**Table 4:** Multivariable logistic regression analysis for the total GIST population with the outcomes anxiety symptoms, depression symptoms and severe FCR.

**Symptoms of anxiety**		Multivariable logistic regression*	
*R² = 0.434*		OR (95% CI)	*p-*value
Sex	Male Female	Reference 2.1 (0.9 – 4.7)	.073
Symptoms of depression	Not present Present	Reference 19.7 (8.2 – 47.2)	**<0.001**
Fear of recurrence or progression	Low fear Severe fear	Reference 2.9 (1.2 – 6.9)	**.016**
Concerns about the need for TKI treatment in the future	No Yes	Reference 2.5 (1.1 – 5.6)	**.031**
*The results of the full univariable logistic regression analysis are available as supplementary table A1, here we only report the multivariable logistic regression.			
**Symptoms of depression**		Multivariable logistic regression*	
***R²*** ***=*** ***0.416***		**OR (95 % CI)**	***p-*****value**
Currently on TKI	No Yes	Reference 3.2 (1.3 – 7.5)	**.009**
Symptoms of anxiety	Not present Present	Reference 24.2 (10.3 – 56.9)	**<0.001**
Concerns about dying from GIST in the long run	No Yes	Reference 2.4 (0.9 – 6.2)	.069
*The results of the full univariable logistic regression analysis are available as supplementary table A2, here we only report the multivariable logistic regression.			
**Severe fear of recurrence or progression**		Multivariable logistic regression*	
***R²*** ***=*** ***0.510***		**OR (95 % CI)**	***p-*****value**
Sex	Male Female	Reference 2.2 (1.2 – 4.1)	**.012**
Age at survey completion		.95 (0.92 - 0.98)	**.001**
Treatment setting	Curative Palliative	Reference 2.3 (1.1 – 5.1)	**.032**
Symptoms of anxiety	Not present Present	Reference 3.8 (1.5 – 9.9)	**.006**
Concerns about the need for TKI treatment in the future	No Yes	Reference 2.4 (1.3 – 4.4)	**.008**
Concerns about dying from GIST in the near future	No Yes	Reference 4.4 (2.1 – 9.3)	**<0.001**
Concerns about dying from GIST in the long run	No Yes	Reference 3.0 (1.5 – 6.4)	**.003**
*The results of the full univariable logistic regression analysis are available as supplementary table A3, here we only report the multivariable logistic regression.			

### Impact on HRQoL

**Fig. 1:**
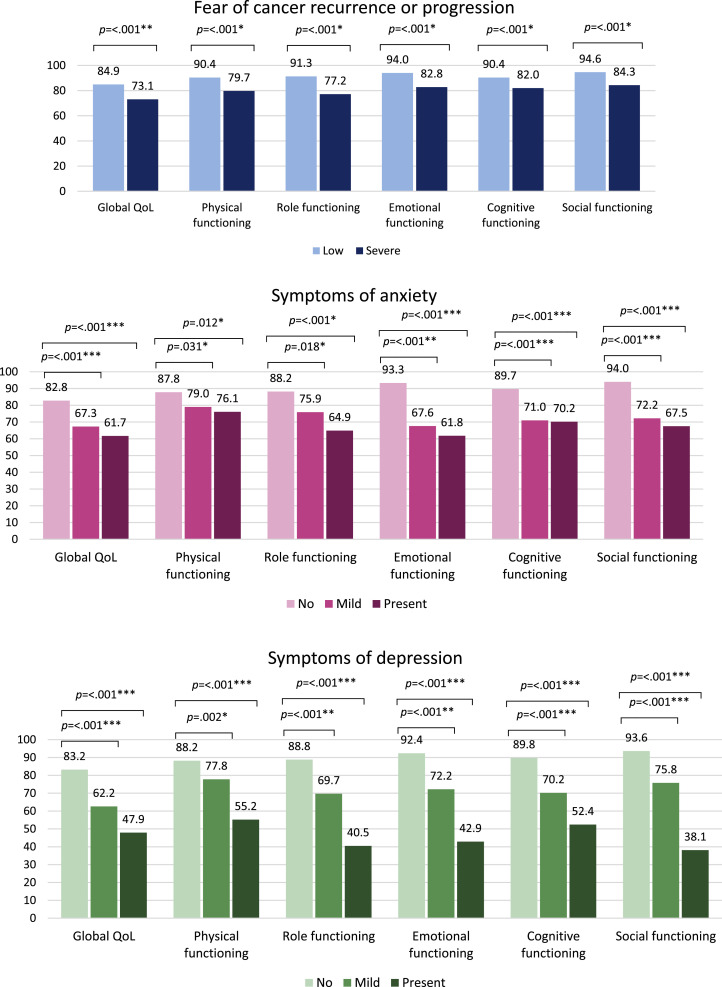
Comparison of mean scores on global QoL and functioning scales of the EORTC QLQ-C30 among patients with low and severe FCR, and patients with no, mild and present symptoms of anxiety and depression – on global QoL and functioning scales, higher scores indicate a better global quality of life and functioning. The mean differences between both groups were considered *small, **medium or ***large, when referring to clinical relevance (Cocks et al., 2012*).*

Experiencing severe FCR resulted in a significant impaired global QoL and physical, role, emotional, cognitive and social functioning, when compared to patients experiencing low FCR (Fig. 1). GIST patients with severe FCR also reported significantly more symptoms of fatigue, pain, dyspnea, insomnia, loss of appetite, nausea and vomiting, diarrhea, and financial difficulties in comparison to patients with low fear (Supplementary table B). Compared to patients with no symptoms of anxiety, having mild symptoms led to a significantly impaired global QoL and functioning on all scales, and when having present symptoms, global QoL and functioning were even more impaired. Patients with mild and present symptoms of anxiety had significant higher scores on the symptom scales fatigue, nausea and vomiting, pain, insomnia, and loss of appetite indicating a higher symptom burden. Besides, they experienced significantly more financial difficulties. For patients with mild and present symptoms of depression, a similar pattern resulting in even more impaired global QoL and functioning scores was found, when compared to patients with no symptoms of depression. In patients with mild and present symptoms of depression a higher symptom burden for fatigue, pain, dyspnea, insomnia, diarrhea, and financial difficulties was reported.

## Discussion

In this cross-sectional study we investigated psychological symptoms of anxiety, depression and FCR among GIST patients. Of the 328 patients, 15% reported anxiety symptoms, 13% depression symptoms, and 43% had severe FCR. Significantly more GIST patients in the palliative setting suffered from these psychological symptoms, as in this group 22% had anxiety symptoms, 26% symptoms of depression, and 72% severe FCR, respectively. In comparison to the norm population, anxiety and depression levels were comparable between the norm population and patients in the curative setting, but significantly higher for patients in the palliative setting.

Several studies that assessed anxiety and depression in large samples of cancer patients with various types of cancer and in different stages, reported similar prevalence rates of 12% to 25% (Brintzenhofe-Szoc et al., 2009; Linden et al., 2012). Few studies investigated anxiety and depression in GIST patients. In a study among Mexican GIST patients, 31% of the 89 patients experienced psychological distress, which was associated with higher levels of fatigue, lower quality of life and functioning (Carbajal-López et al., 2022). In a German study, 22% of the GIST patients experienced anxiety or depression (Eichler et al., 2022). Moreover, in this study GIST and other sarcoma patients were analyzed together, showing disabled persons, patients in precarious employment, newly diagnosed patients and those with progressive disease should be considered as vulnerable groups for developing anxiety or depression.

In our study, having symptoms of depression, concerns and fears were associated with higher odds of anxiety symptoms, suggesting that these psychological symptoms often occur together as clusters. For depression symptoms, besides symptoms of anxiety, being currently on TKIs was associated with higher odds. That current treatment with TKIs contributes to depression symptoms could depend on multiple factors. It could be a direct side effect of imatinib, or a result arising from all experienced side effects, or due to the greater doubts and uncertainties that patients on TKIs experience (e.g., will the treatment be effective, and more in a palliative setting, for how long will treatment be effective). Depression as a direct side effect of imatinib is not described in the literature over the past years, while we have over 20 years of experience with imatinib nowadays. A more likely explanation is that depression symptoms are consequences arising from all experienced side effects of imatinib, and that there is an overlap in the items of the HADS regarding depression symptoms and these consequences. Although the HADS items are about feelings and moods, they are also about looking forward and enjoying social activities or hobbies (Olssøn et al., 2005), this can be influenced by side effects of imatinib and other TKIs, as was described previously (Fauske et al., 2020; van de Wal et al., 2023). In general, imatinib is described as tolerable compared to other systemic therapies, such as chemotherapy. However, this has to be placed into a broader perspective. Chemotherapy results in acute and short term side effects, while TKIs result in less severe, but daily and long-lasting side effects. In particular patients in the palliative treatment setting are depending on TKIs and have to continue treatment, and therefore have to cope with these side effects every day. The continuous fatigue and unexpected diarrhea, both common side effects of TKIs (van de Wal et al., 2022), can make patients less able to enjoy social activities, or more worried when they go out, especially if patients compare this situation to before their treatment.

Severe FCR in GIST patients was a common psychological symptom, present in almost three-fourth of the palliative patients, but also in one third of the curative patients. The prevalence of 43% overall was lower compared to that of the study of Custers et al. (2015), where 52% of the patients with localized or metastatic GIST reported severe FCR. This difference is possibly explained by the fact that less patients were on current TKI treatment (34%) in our study compared to the study of Custers et al. (61%), and part of our sample was considered cured without being in follow-up any longer. As frequent CT scans and follow-up consultations represent a constant reminder of the cancer and risk of recurrence or progression, these patients are less exposed to these triggers and therefore experience less FCR (Bui et al., 2022; Custers et al., 2021). Other studies that used the CWS to asses FCR reported severe FCR in 31% of the breast cancer patients (Custers et al., 2015), 35% of the prostate cancer patients (van de Wal et al., 2017), 38% of the colorectal cancer patients (Custers et al., 2016), and 45% of the young sarcoma survivors (Pellegrini et al., 2022). Previous studies reported that FCR can be found for all time periods since the cancer diagnosis, but that females, higher number of comorbidities and multimodal treatment were associated with a higher risk, whereas aged decreased this risk (Luigjes-Huizer et al., 2022; Pellegrini et al., 2022). This was partly in line with our study, were being female and receiving treatment in a palliative setting were associated with higher odds of severe FCR, and older age with lower odds.

Our findings regarding diminished quality of life and functioning, and higher symptom burden in patients with psychological symptoms were consistent with previous studies (Arrieta et al., 2013; Carbajal-López et al., 2022). Fatigue was one of the symptoms that was more severely present among patients with psycholocial symptoms as they reported significant higher scores of fatigue than patients without psychological symptoms. Fatigue and its impact were also studied in a Dutch sample of GIST patients. In this sample, 30% of the GIST patients suffered from severe fatigue resulting in higher levels of psychological distress, and impaired quality of life and functioning (Poort et al., 2016). It remains unclear if the higher symptom burden is a result of the psychological symptoms, considering that depression and severe FCR were associated with current TKI treatment and a palliative treatment setting, therefore the higher symptom burden can also be a result of the TKI treatment or GIST itself.

Considering the significant impact of psychological symptoms on HRQoL, the HRQoL of patients can be improved if psychological symptoms are recognized and the follow-up steps are clear. Psychological symptoms are sometimes difficult to identify for surgeons and oncologists, and at the same time there is a barrier in referring patients to psychological or psychiatric care (Keller et al., 2004; Passik et al., 1998). This was underlined by a study in which 73% of the cancer patients remained untreated for their depression, merely 24% received an antidepressant and only 5% were seen by a mental health specialists (Walker et al., 2014). This year a European Society for Medical Oncology (ESMO) clinical practice guideline for anxiety and depression in adult cancer patients was published (Grassi et al., 2023). It is recommend to regularly screen for psychological symptoms. In case of GIST, both the HADS and CWS can be used. However, these tools are not sufficient to diagnose anxiety or depression disorders. Thus, when scored above the cut-off value, clinicians and oncology nurses should follow up on this and refer patients for a more formal assessment by a trained expert in psychology, to determine if specialized help or psychological treatment is required. If indicated, patients could benefit from psychoeducation, supportive therapy, cognitive-behavioral therapy, relaxation training, mindfulness-based therapy, or treatment with antidepressants (Carbajal-López et al., 2022; Grassi et al., 2023; Lyu et al., 2022). As around half of the patients declines specialized help, and only one in four patients accepts referrals to psychological care (Tondorf et al., 2018), there is still a lot to be gained. What clinicians and oncology nurses could do is motivate patients to participate in screening and to be referred if indicated, and on the other hand try to reduce possible triggers for psychological symptoms. For instance, reduce the frequency of CT or MRI scans in GIST patients with a long-time stable disease, so patients suffer less from scan-related anxiety, fears and emotional distress before and after these evaluation moments.

To the best of our knowledge, this is the largest sample of GIST patients in which psychological symptoms were studied. Our study population was diverse, including GIST patients on TKIs in a palliative and curative treatment setting, but also patients that survived GIST, with some not even being in follow up any longer. This resulted in a representative cohort of GIST patients in different stages of treatment and follow-up, which made it possible to draw conclusions for the total group of GIST patients, but also possible to analyze subgroups that are more prone for psychological symptoms such as patients in a palliative setting. Our study had several limitations. First, the cross-sectional design limited us to study causalities and changes over time. Second, this was a multicenter study conducted in the Netherlands, therefore only Dutch GIST patients were included, which could impede the generalizability. Last, since reasons for not participating in this study were not collected, and could be due to either poor (mental) health or absence of symptoms, there could be some selection bias.

## Conclusion

In conclusion, the prevalence of anxiety and depression symptoms in palliative treated GIST patients is higher compared to the norm population, while the prevalence in curatively treated patients was comparable with the norm population. Given the relatively high prevalence of psychological symptoms and their considerable impact on the patients’ HRQoL, particularly in palliative GIST patients, this deserves more attention in clinical practice. Through regular screening, these symptoms can be recognized and patients can be offered appropriate interventions.

## Funding

This study was partly funded by research grant from 10.13039/100004336Novartis (grant 006.18). The funder had no role in the design and conduct of the study; in the collection, analyses, or interpretation of data; in the writing of the manuscript; or in the decision to publish the results.

## Author contributions

I.D, O.H and W.G contributed to the study conception, design and methodology. Data curation was done by D.H, I.D, H.G, A.O, A.R, N.S, O.H and W.G. Formal analysis were performed by D.W, under the supervision of O.H and W.G. Funding acquisition was done by I.D., O.H. and W.G. The visualization and original draft of the manuscript was written by D.W. All authors reviewed and edited the draft version of the manuscript, and approved the final manuscript.

## Ethics approval

This study was performed in line with the principles of the Declaration of Helsinki. Ethical approval was obtained from the medical ethical committee of the Radboud University Medical Center (2019-5888). According to the medical ethical regulations, approval of one ethical committee for survey research is valid for all participating centres.

## Consent to participate

Written informed consent was obtained from all individual patients included in the study.

## Declaration of competing interest

The authors declare that they have no known competing financial interests or personal relationships that could have appeared to influence the work reported in this paper.

## Appendix Supplementary materials

Image, application 1
