# Supplementary material for: Fear, anxiety and depression in gastrointestinal stromal tumor (GIST) patients in the Netherlands: Data from a cross-sectional multicenter study
Source: Int J Clin Health Psychol. 2024 Jan 9;24(1):100434. doi: 10.1016/j.ijchp.2023.100434 (PMC10788803; doi:10.1016/j.ijchp.2023.100434)
Supplement: Supplementary file 1 [file mmc1.docx]

Supplementary table A1. Univariable logistic regression analysis for the total GIST population with the outcome symptoms of anxiety, depression symptoms and severe FCR.

| Symptoms of anxiety | | Univariable logistic regression | |
| --- | --- | --- | --- |
|  | | OR (95% CI) | *p-*value |
| Sex | Male  Female | Reference  1.8 (0.9-3.3) | **.078** |
| Age at survey completion | | 1.0 (1.0-1.0) | .586 |
| Socio-economic status | High  Low | Reference  0.8 (0.4-1.5) | .534 |
| Marital status | Married / Living with partner  Not living with a partner | Reference  2.0 (1.0-3.8) | **.050** |
| Educational level | High  Low/intermediate | Reference  2.1 (1.0-4.2) | **.048** |
| Comorbidity | None  1  ≥2 | Reference  1.5 (0.5-4.0)  2.7 (1.2-6.0) | .460  **.014** |
| Time since diagnosis in years | | 1.1 (1.0-1.2) | .201 |
| Location primary GIST | Stomach  Other than stomach | Reference  1.4 (0.7-2.6) | .335 |
| Treatment setting | Curative  Palliative | Reference  1.8 (0.9-3.7) | **.086** |
| Had surgery for GIST at some point | No  Yes | Reference  1.4 (0.4-5.0) | .559 |
| Received TKI at some point | No  Yes | Reference  1.5 (0.7-2.9) | .273 |
| Currently on TKI | No  Yes | Reference  1.5 (0.8-2.8) | .223 |
| Symptoms of depression | Not present  Present | Reference  24.4 (11.1-53.7) | **<.001** |
| Fear of recurrence or progression | Low fear  Severe fear | Reference  6.4 (3.1-13.5) | **<.001** |
| Concerns about the need for TKI treatment in the future | No  Yes | Reference  3.9 (2.0-7.5) | **<.001** |
| Concerns about dying from GIST in the near future | No  Yes | Reference  3.9 (2.0-7.5) | **<.001** |
| Concerns about dying from GIST in the long-term future | No  Yes | Reference  3.1 (1.5-6.2) | **.002** |

Supplementary table A2. Univariable logistic regression analysis for the total GIST population with the outcome symptoms of depression.

| Symptoms of depression | | Univariable logistic regression | |
| --- | --- | --- | --- |
|  | | OR (95% CI) | *p-*value |
| Sex | Male  Female | Reference  0.9 (0.5-1.8) | .821 |
| Age at survey completion | | 1.0 (0.9-1.0) | .150 |
| Socio-economic status | High  Low | Reference  0.9 (0.4-1.7) | .666 |
| Marital status | Married / Living with partner  Not living with a partner | Reference  1.5 (0.7-3.2) | .250 |
| Educational level | High  Low/intermediate | Reference  1.4 (0.7-2.9) | .371 |
| Comorbidity | None  1  ≥2 | Reference  0.8 (0.3-2.4)  2.1 (1.0-4.6) | .654  **.062** |
| Time since diagnosis in years | | 1.1 (1.0-1.2) | .201 |
| Location primary GIST | Stomach  Other than stomach | Reference  1.6 (0.8-3.1) | .154 |
| Treatment setting | Curative  Palliative | Reference  3.4 (1.7-6.8) | **<.001** |
| Had surgery for GIST at some point | No  Yes | Reference  0.5 (0.2-1.3) | .147 |
| Received TKI at some point | No  Yes | Reference  3.5 (1.4-8.7) | **.006** |
| Currently on TKI | No  Yes | Reference  3.1 (1.6-6.1) | **<.001** |
| Symptoms of anxiety | Not present  Present | Reference  24.3 (11.1-53.7) | **<.001** |
| Fear of recurrence or progression | Low fear  Severe fear | Reference  5.9 (2.7-12.9) | **<.001** |
| Concerns about the need for TKI treatment in the future | No  Yes | Reference  2.6 (1.3-5.1) | **.005** |
| Concerns about dying from GIST in the near future | No  Yes | Reference  4.8 (2.3-9.9) | **<.001** |
| Concerns about dying from GIST in the long-term future | No  Yes | Reference  4.4 (2.0-9.9) | **<.001** |

Supplementary table A3. Univariable logistic regression analysis for the total GIST population with the outcome severe FCR.

| Severe fear of recurrence or progression | | Univariable logistic regression | |
| --- | --- | --- | --- |
|  | | OR (95% CI) | *p-*value |
| Sex | Male  Female | Reference  1.6 (1.0-2.5) | **.034** |
| Age at survey completion | | 1.0 (0.9-1.0) | **.007** |
| Socio-economic status | High  Low | Reference  1.0 (0.6-1.6) | .986 |
| Marital status | Married / Living with partner  Not living with a partner | Reference  0.8 (0.4-1.3) | .306 |
| Educational level | High  Low/intermediate | Reference  0.9 (0.6-1.5) | .742 |
| Comorbidity | None  1  ≥2 | Reference  1.1 (0.6-2.2)  2.3 (1.4-3.9) | .705  **.001** |
| Time since diagnosis in years | | 1.1 (1.0-1.1) | .211 |
| Location primary GIST | Stomach  Other than stomach | Reference  1.2 (0.7-1.8) | .510 |
| Treatment setting | Curative  Palliative | Reference  4.6 (2.5-8.5) | **<.001** |
| Had surgery for GIST at some point | Yes  No | Reference  0.6 (0.3-1.3) | .169 |
| Received TKI at some point | No  Yes | Reference  1.8 (1.1-2.9) | **.016** |
| Currently on TKI | No  Yes | Reference  2.5 (1.5-4.0) | **<.001** |
| Symptoms of anxiety | Not present  Present | Reference  6.4 (3.1-13.5) | **<.001** |
| Symptoms of depression | Not present  Present | Reference  5.9 (2.7-12.9) | **<.001** |
| Concerns about the need for TKI treatment in the future | No  Yes | Reference  4.1 (2.5-6.6) | **<.001** |
| Concerns about dying from GIST in the near future | No  Yes | Reference  11.4 (6.6-19.5) | **<.001** |
| Concerns about dying from GIST in the long-term future | No  Yes | Reference  9.7 (5.7-16.5) | **<.001** |

Supplementary table B. Comparison of mean scores on global QoL, functioning scales and symptoms scales of the EORTC QLQ-C30 among patients with low and severe FCR, and patients with no, mild and present symptoms of anxiety and depression.

|  | **FCR** | **Mean ±** **SD** | ***p*-value** | **Anxiety** | **Mean ±** **SD** | **Posthoc**  **Bonferroni** | **Depression** | **Mean ±** **SD** | **Posthoc**  **Bonferroni** |
| --- | --- | --- | --- | --- | --- | --- | --- | --- | --- |
| Global QoL | Low  Severe | 84.9 ± 14.9  73.1 ± 17.0 | **<.001**** | No  Mild  Present | 82.8 ± 15.2  67.3 ± 16.3  61.7 ± 19.2 | **<.001*****  **<.001***** | No  Mild  Present | 83.2 ± 14.7  62.6 ± 15.9  47.9 ± 13.2 | **<.001*****  **<.001***** |
| Physical functioning | Low  Severe | 90.4 ± 14.9  79.7 ± 19.3 | **<.001*** | No  Mild  Present | 87.8 ± 16.0  79.0 ± 20.8  76.1 ± 22.3 | **.031***  **.012*** | No  Mild  Present | 88.2 ± 16.1  77.8 ± 18.1  55.2 ± 12.0 | **.002***  **<.001***** |
| Role functioning | Low  Severe | 91.3 ± 18.7  77.2 ± 25.8 | **<.001*** | No  Mild  Present | 88.2 ± 20.4  75.9 ± 29.0  64.9 ± 30.9 | **.018***  **<.001*** | No  Mild  Present | 88.8 ± 20.3  69.7 ± 26.2  40.5 ± 23.3 | **<.001****  **<.001***** |
| Emotional functioning | Low  Severe | 94.0 ± 12.6  82.8 ± 18.8 | **<.001*** | No  Mild  Present | 93.3 ± 10.9  67.6 ± 22.3  61.8 ± 23.1 | **<.001****  **<.001***** | No  Mild  Present | 92.4 ± 12.9  72.2 ± 18.5  42.9 ± 18.9 | **<.001****  **<.001***** |
| Cognitive functioning | Low  Severe | 90.4 ± 14.3  82.0 ± 21.3 | **<.001*** | No  Mild  Present | 89.7 ± 14.6  71.0 ± 27.6  70.2 ± 25.2 | **<.001*****  **<.001***** | No  Mild  Present | 89.8 ± 14.6  70.2 ± 23.1  52.4 ± 33.9 | **<.001*****  **<.001***** |
| Social functioning | Low  Severe | 94.6 ± 16.2  84.3 ± 22.0 | **<.001*** | No  Mild  Present | 94.0 ± 14.9  72.2 ± 23.6  67.5 ± 35.3 | **<.001*****  **<.001***** | No  Mild  Present | 93.6 ± 14.7  75.8 ± 28.3  38.1 ± 28.4 | **<.001*****  **<.001***** |
| Fatigue | Low  Severe | 14.7 ± 18.8  29.1 ± 22.6 | **<.001**** | No  Mild  Present | 17.9 ± 19.6  36.6 ± 27.7  33.3 ± 22.5 | **<.001****  **.005**** | No  Mild  Present | 17.1 ± 18.8  39.7 ± 23.4  57.1 ± 28.3 | **<.001*****  **<.001***** |
| Nausea and vomiting | Low  Severe | 3.7 ± 13.0  7.5 ± 16.4 | **.026*** | No  Mild  Present | 3.6 ± 10.3  17.3 ± 30.1  12.3 ± 23.5 | **<.001****  **.029**** | No  Mild  Present | 4.2 ± 12.7  9.1 ± 19.1  28.6 ± 34.3 | .194  **<.001***** |
| Pain | Low  Severe | 7.3 ± 16.5  17.0 ± 23.7 | **<.001*** | No  Mild  Present | 8.1 ± 17.1  24.1 ± 21.8  33.3 ± 34.2 | **<.001****  **<.001***** | No  Mild  Present | 8.5 ± 17.6  27.8 ± 27.5  31.0 ± 29.5 | **<.001*****  **.007***** |
| Dyspnoea | Low  Severe | 8.1 ± 17.5  18.0 ± 24.4 | **<.001**** | No  Mild  Present | 10.4 ± 19.3  21.0 ± 24.7  14.0 ± 16.9 | **.025****  1.000 | No  Mild  Present | 10.2 ± 19.2  19.2 ± 20.5  28.6 ± 30.0 | **.039****  **.044***** |
| Insomnia | Low  Severe | 12.1 ± 22.3  25.4 ± 29.7 | **<.001**** | No  Mild  Present | 13.8 ± 22.9  38.3 ± 31.6  42.1 ± 34.9 | **.001*****  **.007***** | No  Mild  Present | 15.0 ± 24.4  31.3 ± 26.3  57.1 ± 41.8 | **.001****  **<.001***** |
| Loss of appetite | Low  Severe | 4.1 ± 14.4  12.8 ± 23.4 | **<.001*** | No  Mild  Present | 4.9 ± 14.7  25.9 ± 29.7  15.8 ± 25.7 | **<.001****  **.024*** | No  Mild  Present | 5.5 ± 16.4  20.2 ± 24.9  19.0 ± 26.2 | **<.001****  .140 |
| Constipation | Low  Severe | 4.6 ± 13.1  7.4 ± 18.5 | .138 | No  Mild  Present | 4.5 ± 12.8  9.9 ± 20.3  17.5 ± 32.1 | .253  **<.001**** | No  Mild  Present | 5.0 ± 14.4  10.1 ± 21.2  14.3 ± 26.2 | .234  .364 |
| Diarrhoea | Low  Severe | 9.1 ± 20.5  18.5 ± 27.5 | **<.001**** | No  Mild  Present | 11.2 ± 22.0  19.8 ± 31.0  26.3 ± 34.4 | .222  **.023**** | No  Mild  Present | 10.8 ± 22.0  24.2 ± 30.4  38.1 ± 40.5 | **.006****  **.008**** |
| Financial difficulties | Low  Severe | 1.3 ± 8.9  10.1 ± 24.5 | **<.001*** | No  Mild  Present | 2.5 ± 12.0  16.0 ± 28.3  22.8 ± 38.6 | **<.001****  **<.001**** | No  Mild  Present | 2.3 ± 11.4  19.2 ± 32.3  38.1 ± 44.8 | **<.001****  **<.001**** |

On global QoL and functioning scales, higher scores indicate a better global quality of life and functioning. On symptom scales, higher score indicate a higher symptom burden. The mean differences between both groups were considered *small, **medium or ***large*, when referring to clinical relevance (Cocks et al., 2012)*.
